# Financial toxicity among cancer survivors: a conceptual model based on a feedback perspective

**DOI:** 10.1007/s00520-023-08066-x

**Published:** 2023-10-07

**Authors:** Alexander Scheidegger, Daniela Bernhardsgrütter, Andrea Kobleder, Martin Müller, Karen Nestor, Ernst Richle, Eleonore Baum

**Affiliations:** 1 Ostschweizer Fachhochschule, Oberseestrasse 10, 8640 Rapperswil, Switzerland; 2 Kantonsspital St. Gallen, Rorschacher Strasse 95, 9007 St. Gallen, Switzerland; 3 Krebsliga Ostschweiz, Flurhofstrasse 7, 9000 St. Gallen, Switzerland

**Keywords:** Financial toxicity, Cancer, Participatory modeling, Survivorship, Economic impact, Patient perspectives

## Abstract

**Purpose:**

Experiencing financial toxicity following a cancer diagnosis is a circular and complex process. We investigate the circular causal mechanisms that either reinforce or balance financial toxicity dynamics.

**Methods:**

We conducted a literature review, expert interviews, a participatory modeling process, and exploratory interviews with *N* = 11 adults with cancer living in Switzerland. We sampled participants purposively based on health-related and sociodemographic characteristics.

**Results:**

We describe a conceptual model based on the triangulation of cancer survivor narratives, expert perspectives, and a literature review. This model distinguishes between the reinforcing and balancing feedback loops that drive the dynamics of financial toxicity. It includes the topics “Coping with cancer and employment,” “Coping with limited economic resources,” and “Maintaining care resources while facing economic pressure.” For each topic, we identify a necessary condition for cancer survivors to avoid reinforcing financial toxicity.

**Conclusions:**

The results allow us to reconstruct participant narratives regarding cancer-related financial toxicity. Based on comparison with scientific literature from Western Europe and North America, we hypothesize the validity of the model beyond the population covered by the sample. The results highlight the importance of screening for the risk of financial toxicity in the clinical context and individual risk and resource assessment in social counseling.

**Implications for cancer survivors:**

These results can raise cancer survivors’ awareness of risks related to financial toxicity and strengthen their resources for coping with financial burden successfully.

**Supplementary Information:**

The online version contains supplementary material available at 10.1007/s00520-023-08066-x.

## Introduction

Cancer survivors and their families face physical, psychosocial, and practical challenges caused by the disease and its treatment [1]. These challenges may occur throughout the entire course of cancer—even before the actual diagnosis and after the completion of treatment. One challenge is the possible deterioration of the economic situation of patients and their families that can be associated with a cancer diagnosis. Previous studies, which have been conducted mainly in the USA, reveal that nearly half of all cancer survivors experience financial distress [2]. This distress can be described by the term “financial toxicity” [3].

It is known that the financial burden faced by cancer survivors and their families results from both the direct and indirect costs of the disease. Direct costs may be medical (e.g., out-of-pocket payments for medications, medical devices, and insurance costs) or nonmedical (e.g., the costs of housekeeping in cases in which some activities cannot be performed by cancer survivors). The most significant factor influencing difficult economic circumstances, however, is the indirect costs, for example, those resulting from a necessary reduction in the cancer survivor’s level of employment, job loss, or late reimbursements by insurers [4, 5]. These factors are associated with an increased risk of poverty [6]. Moreover, several preexisting circumstances are associated with a greater risk of financial toxicity, such as male gender, younger age, low education, low socioeconomic status, and no paid employment [7].

Fitch et al. [5] investigated the experiences of patients and their families with the financial burden associated with cancer. Their synthesis of qualitative studies provides a holistic perspective on the ways in which cancer-related financial burden arises. In addition, their research provides insight into the strategies used by cancer survivors and their families to mitigate the impact of financial hardship. Experiencing financial toxicity following a cancer diagnosis was found to be a circular and complex process: “Individuals’ pre-existing circumstances, including available support from family and friends, and their responses at various points throughout the cancer experience influence the perception of financial hardship, the capacity to cope, and the subsequent impact on quality of life. Clearly, this can continue to change over time” (p. 320).

Cancer-related financial distress has a multifaceted impact on cancer survivors [8] and is associated with the emotional distress experienced by cancer survivors and their family members, which is associated with lower quality of life [9, 10]. Cancer survivors and/or their family members experience anger; fears regarding the future, including existential concerns; and guilt for being the reason for financial burden and its consequences [5]. Financially induced anxiety and stress, in turn, can affect cancer survivors’ health status in the long term. Pre- and postdiagnosis financial stress has been associated with a higher risk of cancer-related fatigue [11]. Moreover, initial evidence has suggested that financial burden may also lead to an increased risk of cancer recurrence and mortality and to noncompliance on the part of cancer survivors concerning medication, treatment recommendations, or medical consultations [12, 13].

In summary, cancer-related impacts on financial burden and its consequences for cancer survivors and their families can be described as multifaceted. The term cancer survivors, in this context, refers to “all individuals having been diagnosed with and treated for cancer, either living with or free of cancer [14].” This scope is appropriate for the question at hand since the financial consequences of cancer start at the time of diagnosis or even earlier. Moreover, several impact factors interact with one another, and these impacts may change over time and can thus have delayed effects on economic resources; in some cases, effects can be observed decades after the cancer diagnosis [15]. However, the ways in which specific impact factors influence each other within the circular process of financial toxicity remain unknown.

Some cancer survivors are able to stabilize the multiple cancer-related impacts on their financial situation by using coping strategies and adapting to their changed situation. They can balance their economic situation in a way that allows them to reduce undesirable effects to ensure that their financial situation does not become toxic. Accessing social support or becoming financially frugal and proactive (e.g., by reducing expenses through lifestyle changes) is possible coping strategies that may have a balancing effect on the economic situation of cancer survivors [16]. However, in other cases, initial disturbances trigger a downward spiral, such that the initial impact on the financial situation triggers a reinforcing dynamic involving not only economic resources but also social relationships and support, emotional and physical burden or earning/career capabilities [17, 18]. To assess the possible effects of interventions, it is necessary to understand these balancing and reinforcing processes clearly and to make distinctions among them.

In this publication, we present a conceptual model for the behavior over time of financial toxicity associated with cancer. This model explicitly adopts a feedback perspective [19]. Accordingly, both balancing and reinforcing causal mechanisms that drive financial toxicity over time are explicitly described and distinguished. The boundaries of the model are drawn causally—variables that interact with cancer-related financial toxicity are included in the model.

## Methods

For the purposes of our study, we used a participatory system dynamics [18] modeling approach. System dynamics modeling applies to complex, dynamic problems involving circular, delayed, and accumulating causalities [19].

### System dynamics modeling

This approach has its methodological roots in servo-mechanical engineering. Controlling the state of a dynamic system in an engineering context involves a sensory system that observes the state of the system in question. The signal is processed in a controller that decides on regulatory action, thereby altering a state. Servo-mechanical systems thus involve delayed circular causalities. To design controllers that fulfill the desired purpose, the dynamic system is represented by both graphical (qualitative) and formal (quantitative) models. Since the 1950s, this approach has been adopted in the social sciences to investigate complex dynamic processes that require humans to make decisions and interact with their socioecological environment [19]. In this article, we use a causal loop diagram [20]—a graphical representation of circular causal structures—to represent our findings on the topic of cancer-related financial toxicity.

A causal loop diagram is a graphical model that uses a specific syntax that is capable of representing hypotheses concerning systemic mechanisms involving circular causalities. This model is widely used to reconstruct puzzling dynamics, for example, in systems involving environmental health issues, human–environment interactions, or psychosocial interactions [21]. Its purpose is to represent a so-called causal structure that can be used to understand why a system exhibits a particular dynamic behavior over time [20].

#### Syntax of causal loop diagrams

A causal loop diagram consists of variables that are connected by arrows called causal links (see subsection “Model description” for an  example). A causal link connects a cause to an effect. It can either have a positive ( +) or a negative ( −) polarity. The polarity indicates the manner in which the effect depends on the cause—assuming that all other variables are constant [20]. A positive polarity indicates that the effect changes in the same direction as the cause; a negative polarity indicates that it changes in the opposite direction. Directed cycles in the causal loop diagram are called feedback loops. Feedback loops can either have a balancing (shown as B1, B2… in subsection “Model description”) or a reinforcing (R1, R2…) effect on external changes depending on the number of arrows with a negative polarity that they involve. Directed cycles featuring an odd number of arrows with negative polarity are called balancing feedback loops, while those with an even number of arrows with a negative polarity are called reinforcing feedback loops [20].

#### A constructivist perspective

We used a causal loop diagram to structure ideas regarding why some cancer survivors can stabilize their financial situation while others are driven deeply into financial toxicity by a reinforcing downward spiral. Some readers might perceive a causal loop diagram as a representation of a mechanistic worldview—due to its roots in servo-mechanical engineering—and thus as representing a positivist view of the social sciences. However, in the history of system dynamics practice, causal loop diagrams have been used with both a rather positivist and a rather interpretivist perspective on the social sciences [22]. We have adopted the approach of mapping subjective causal explanations for financial toxicity dynamics from different perspectives and triangulating them. Thus, we consider the structures driving financial toxicity to be intersubjectively constructed. Simultaneously, the subjective interpretation of these structures plays an important role in this process.

### Study design

From a methodological perspective, our research approach was structured into a series of four steps, resulting in a participatory model design process [23] that included different perspectives on financial toxicity.

#### Literature review and expert interviews

In the first step, our interdisciplinary research team conducted a review of the literature on financial toxicity. Relevant studies on this topic in English and German were identified by consulting several databases (PubMed, Cochrane, CINAHL, and PsycINFO). In a second step, we conducted *N* = 5 expert interviews with experienced professionals. These professionals had at least 3 years of experience in counseling cancer survivors with respect to cancer-related financial toxicity. The sample was selected heterogeneously with respect to organizational affiliation, organizational focus (medical care, social counseling, or social insurance), and professional background (social work, oncology nursing, or medicine).

#### Participatory modeling process

As a third step, we implemented a participatory modeling process involving four model design workshops. Two of these workshops were intended for members of the interdisciplinary research team and led by the modeling expert (i.e., all participants were coauthors of this article, AS was the modeling expert, and the coauthors DB, AK, MM, and EB were the participants). In these workshops, we used well-known participatory modeling workshop scripts [24] to integrate the findings from both the expert interviews and the literature review into a first model draft.

Two subsequent workshops were conducted by the research team and the participating experts (*N* = 6, the inclusion criteria were the same as in the expert interviews, the workshops included overlapping samples, and the experts included the coauthors KN and ER). This participatory modeling process resulted in a second draft of the causal loop diagram, which synthesized ideas regarding the essential causal mechanisms underlying the process of balancing and reinforcing cancer-related financial toxicity as described in the scientific literature and observed in expert practice.

#### Empirical validation

In the fourth step, we conducted *N* = 11 exploratory patient interviews [25] to validate the causal loop diagram. We considered a heterogeneous sample of six women and five men. We included adults with cancer and their relatives who were receiving care from one of the participating oncology services. Cancer survivors were excluded if the cancer was diagnosed after the legal retirement age, if they suffered from cognitive impairment, or if they were in a terminal stage of the disease.

Participants were recruited through gatekeepers (nurses, oncologists, and social workers) working in two oncology departments and several social counseling services focused on caring for persons with cancer in the eastern part of Switzerland. The research team purposively sampled participants based on their characteristics, which were provided by the gatekeepers [25]. For the sampling, we considered health-related and sociodemographic characteristics (time since/age at the time of cancer diagnosis, comorbidities, state of residence, employment and financial resources, social insurance benefits, household size and family situation, and the availability of supportive relatives).

The interviewees were aged between 30 and 58 years, and the majority of interviewees were single or divorced; most interviewees were Swiss nationals. All interviewees had adjusted their level of employment during the course of the disease or at the time of diagnosis. Four interviewees had more than one child. In the interviews, the participants reconstructed their narratives concerning the financial issues related to cancer. After the interviews, we compared each participant narrative with the model from the participatory process. We compared each causal assumption underlying the participant narratives with the model. While some of the assumptions could be subsumed under mechanisms that were already represented in the model, others could not. The research team processed the model to best match both the ideas drawn from the participatory modeling process and those drawn from the participants’ perspectives. We could remove some contradictions in the model by redefining variables, while other contradictions required rephrasing or even the addition of new mechanisms following the interviews. However, each individual causal link and each feedback mechanism [26] were included in the model only if its relevance for financial toxicity was confirmed in terms of the following three criteria: (1) theoretical support from the literature on financial toxicity; (2) review by the group of experts, i.e., a mechanism was only included in the model if it was crucial to the task of explaining financial toxicity according to at least one expert’s case narratives and it was not in contradiction to any of the experts’ case narratives; and (3) empirical evidence, in the sense that each mechanism was found in the narrative reconstruction of financial toxicity for at least one participant and was not in contradiction to any of the participants’ case narratives.

## Results

**Fig. 1:**
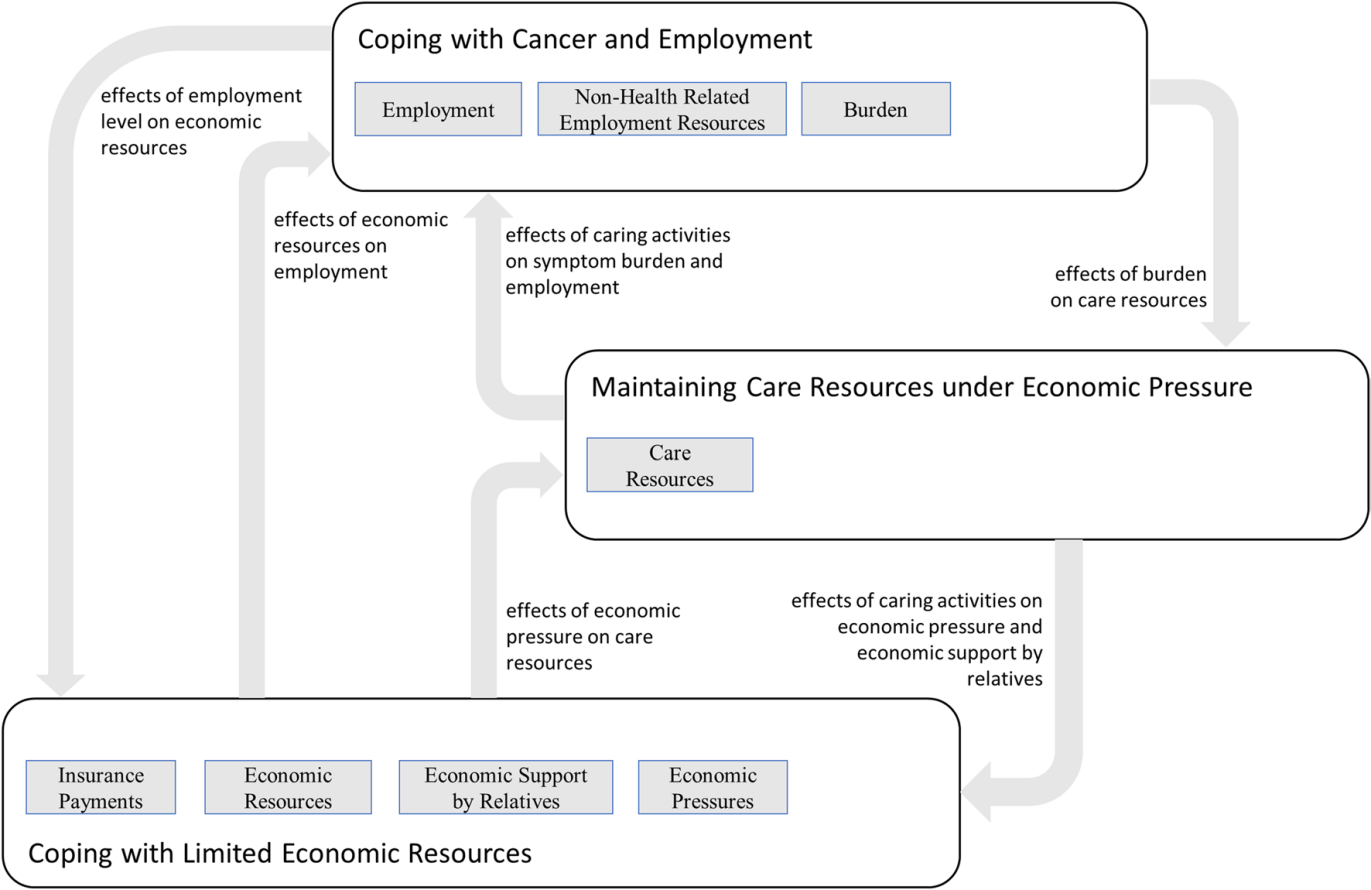
Sector diagram showing relationships between coping with economic resources, with cancer and employment, and maintaining care resources

The resulting causal loop diagram can be found in the supplementary information. In the following “Model description” section, we visualized each sector of the diagram in three figures that are each explained in the three subsections (“Coping with cancer and employment,” “Coping with limited economic resources,” and “Maintaining care resources under economic pressure and burdens”). The interactions between the three sectors are visualized in Fig. 1. In the “Model interpretation” section, we interpret the model, thereby identifying three necessary conditions for cancer survivors to avoid reinforcing financial toxicity.

### Model description

While all substructures of the model potentially interact with one another, we structured the explanation into three parts, focusing on “Coping with cancer and employment,” “Coping with limited economic resources,” and “Maintaining care resources under economic pressure.” These subsections include quotations from the interviews to illustrate each causal relationship and to show how the ideas underlying the model were expressed by interviewees. The causal links and feedback loops alongside the associated explanations refer to behavior over time of financial toxicity. We consider the ways in which, according to the interview participants and experts, a given model element is linked to either balancing or reinforcing the dynamics of financial toxicity. Note that the notions printed in *italics* in this section refer to the variables included in the figures.

#### Coping with cancer and employment

**Fig. 2:**
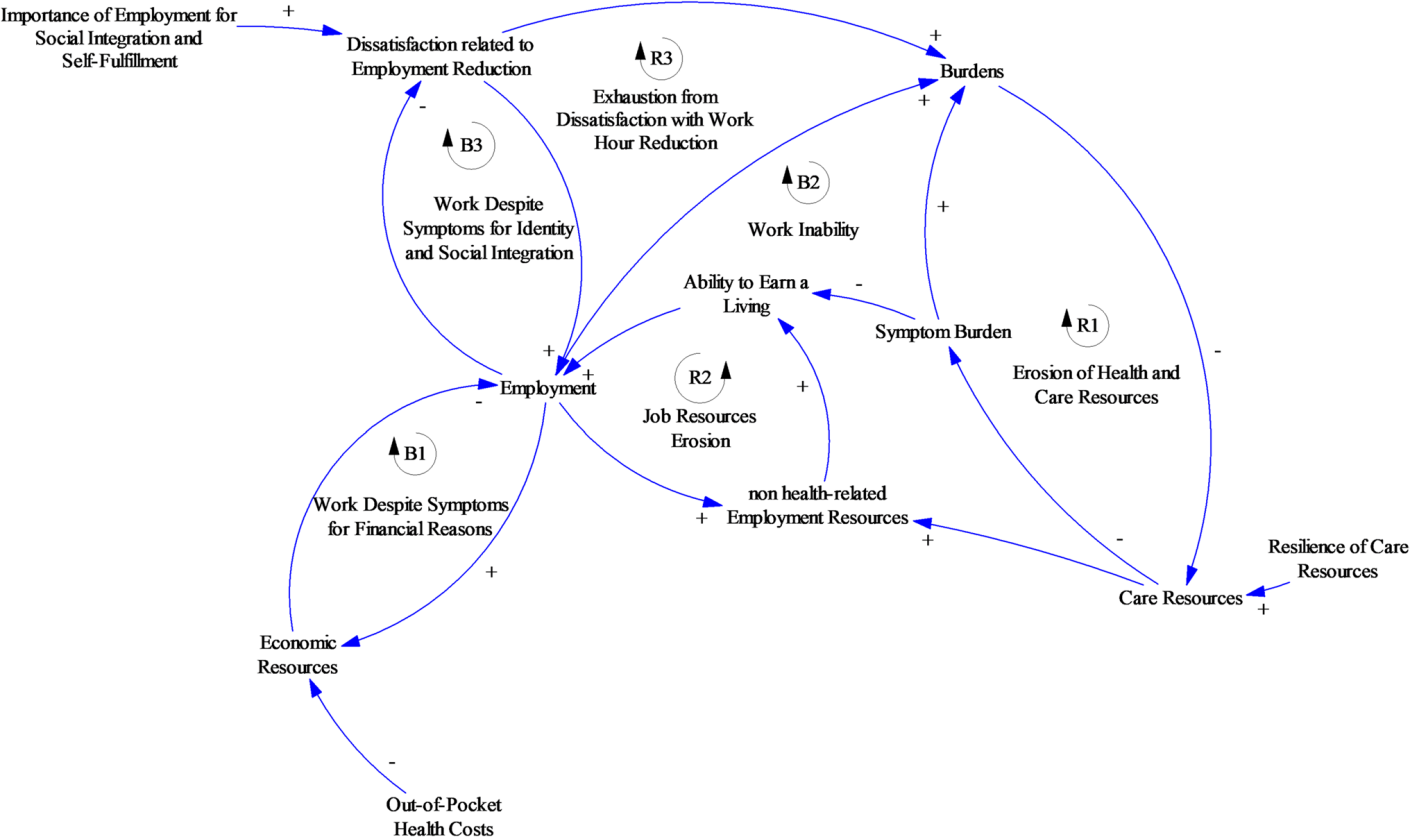
Coping with cancer and employment

The first model sector, which is presented in Fig. 2, focuses on interactions between the symptom burden resulting from cancer and the cancer survivor’s employment, which can also impact his or her financial situation.

 Let us assume a situation in which *Symptom Burden* related to cancer or cancer treatment reaches a level at which the cancer survivor’s *Ability to Earn a Living* is reduced at least temporarily (causal link with a—sign; causality with polarity of the “opposite direction”; higher symptom burden leads to lower *Ability to Earn a Living*, assuming that all other influences remain unchanged). Therefore, the level of *Employment* tends to decrease as well. However, the model accounts for various other factors that influence the level of *Employment* dynamically in addition to *Symptom Burden* and the *Ability to Earn a Living*.

At first, study participants reported maintaining or increasing their *Employment* to a higher level than the one indicated by their health state because reductions in *Employment* constrain their *Economic Resources*. Referring to Fig. 2, less *Employment* entails less *Economic Resources*; less *Economic Resources*, however, imply that the level of *Employment* is higher than it would be otherwise. This situation refers to the two causal links between the variables *Employment* and *Economic Resources*. Two arrows (one with a + polarity and one with a − polarity) form the balancing feedback loop *B1*. Assuming that all the external influences on the variables in this feedback loop remain unchanged, any external impact reducing the level of *employment* tends to be balanced by the feedback loop B1. Two patient narratives illustrating this feedback loop are presented in Table 1.

**Table 1:** Narratives for coping with cancer and employment

Model relationships (Fig. 2)	Relevant feedback loop	Participant narrative
*Economic Resources *→* Employment *→* Economic Resources*	*B1—Working Despite Symptoms for Financial Reasons*	One participant aimed to increase her level of employment following chemotherapy to secure more financial resources for her family abroad as well as to pay off her debts. She made this attempt despite facing high symptom burden in the form of fatigue (ID4, female, 41 years)
*Economic Resources *→* Employment*	*B1—Working Despite Symptoms for Financial Reasons*	Another participant who had his own business suffered from permanent limitations due to cancer during his childhood. He noted that he did not have the financial resources to afford to work fewer hours and made the following statement: “It’s just difficult because I can already do less than a healthy person, and then even less … then it gets really tight really fast” (ID01, male, 40 years)
*Symptom Burden *→ →* Ability to earn a living *→* Employment *→* Burden *→* Care Resources *→* Symptom Burden* *Symptom Burden *→* Burdens *→* Care Resources *→* Symptom Burden*	*B2 – Work Inability, R1 – Erosion of Health and Care Resources*	One participant with breast cancer who suffered from ongoing symptom burden due to chemotherapy feared losing her job and the resulting economic consequences. This fear led her to start working again as soon as possible. She described increasingly severe symptoms, especially fatigue and signs of depression, as well as an increasing discrepancy between her *Ability to Earn a Living* and her *Level of Employment*. The downward spiral R1 led to overburden, which was followed by months of sick leave (ID03, female, 53 years)
*Symptom Burden *→*Burdens *→* Care Resources *→* Symptom Burden*	*R1 – Erosion of Health and Care Resources*	For another participant, the symptoms of fatigue led him to be less able to invest time and energy in his social network, causing him to become more isolated. This isolation caused him to receive less support from friends, family, or colleagues, which once again increased the symptom burden he faced due to exertion in everyday life and loneliness (ID01, male, 40 years)
*Symptom Burden *→*Burdens *→*Care Resources*	*R1 – Erosion of Health and Care Resources*	One participant was required to surrender his driver’s license due to symptom burden, which limited his mobility in daily life (ID05, male, 56 years)
*Employment *→* Non-Health Related Employment Resources *→* Ability to Earn a Living*	*R2 – Erosion of Job Resources*	As result of overburden, ID03 had to go on months of sick leave. She remained absent from work when the insurance money for her company ceased to be provided, and her contract was terminated. In this example, lower *Employment* causes lower *Non-Health Related Employment Resources* (ID03, female, 53 years)
*Employment *→* Dissatisfaction Related to Employment Reduction *→* Employment*	*B3 – Work Despite Symptoms for Identity and Social Integration*	One self-employed participant tried to return to work as soon as possible as he felt proud to continue working at his job despite his illness, and his job offered him a sense of purpose. He enjoyed interacting with his customers very much (ID06, male, 55 years) One young woman to whom we spoke described returning to work as a way of fulfilling a social obligation more than a result of economic pressure: “It is more of a duty than having the money in mind” (ID08, female, 30 years) Another participant suffered from persistent symptoms since childhood. The possibility of closing his business, however, did not come into question. He identified himself strongly with his job, as demonstrated by the following quotation: “It feels good to know you are working for your wages” (ID01, male, 40 years)

However, not all cancer survivors can maintain *Employment* at a high level, as higher levels of *Employment* can lead to more *Burden*. The variable *Burden* describes cancer survivors’ perceived burden, which is not restricted to physical or psychological symptoms. Increased fatigue reduces the ability to take care of oneself. Requesting external help or reaching out to friends for support in this situation becomes increasingly challenging. Under conditions of high burden, participants described entering a reinforcing spiral *R1* featuring eroding physical, psychological, or social resources, as presented in Table 1.

In the model, we used the variable *Care Resources* to refer to the resources used by cancer survivors and their relatives “to maintain, continue and repair their world so that they can live in it as good as possible. That world includes their bodies, their selves, and their environment, all of which they seek to interweave in a complex, life-sustaining web.” This notion of care is based on the definition provided by Tronto [27]. The participants’ reports shown above exemplify the multifaceted ways in which increased *Burden* contributed to reduced *Care Resources* and the fact that the lack of such resources contributes to increased *Symptom Burden* and thus to increased *Burden*. Taken together, these three links form the reinforcing feedback loop *R1*.

The feedback loop labeled *B2—Work Inability* consists of the causal links that have been described above. B2 is a balancing feedback loop. Taking leave from *Employment* due to *Symptom Burden* can support regeneration and a reduction in *Symptom Burden*, thus increasing the *Ability to Earn a Living* and ultimately increasing the level of *Employment* (Table 1 loop B2)*.*

The variable *Non-Health Related Employment Resources* describes all resources that enable a person to earn a living that are not directly related to health. It includes, for instance, professional qualifications, professional networks, or the person’s roles and responsibilities at his or her job. Two participants to whom we spoke had lost their jobs. Self-employed participants were worried about losing employment resources such as their trusted customers. Reductions in their level of employment were linked to this loss. The reinforcing feedback loop *R2 – Erosion of Job Resources* describes the downward spiral narrated by these participants (Table 1 loop R2).

However, another reason for which participants maintained a high level of *Employment* despite high *Symptom Burden* was the perceived *Importance of Employment for Social Integration and Self-Fulfilment*. For some participants, a reduction in employment caused high levels of *Dissatisfaction Related to Employment Reduction* (R3). This dissatisfaction led them to increase their employment while continuing to face a high level of *Symptom Burden* or even to avoid any reductions in *Employment* (balancing feedback *B3 – Work Despite Symptoms for Identity and Social Integration*). For several participants, the workplace was their primary opportunity to connect with others and an important part of their social lives in addition to a source of financial security, as highlighted by participant narratives in Table 1 illustrating loop B3.

#### Coping with limited economic resources

**Fig. 3:**
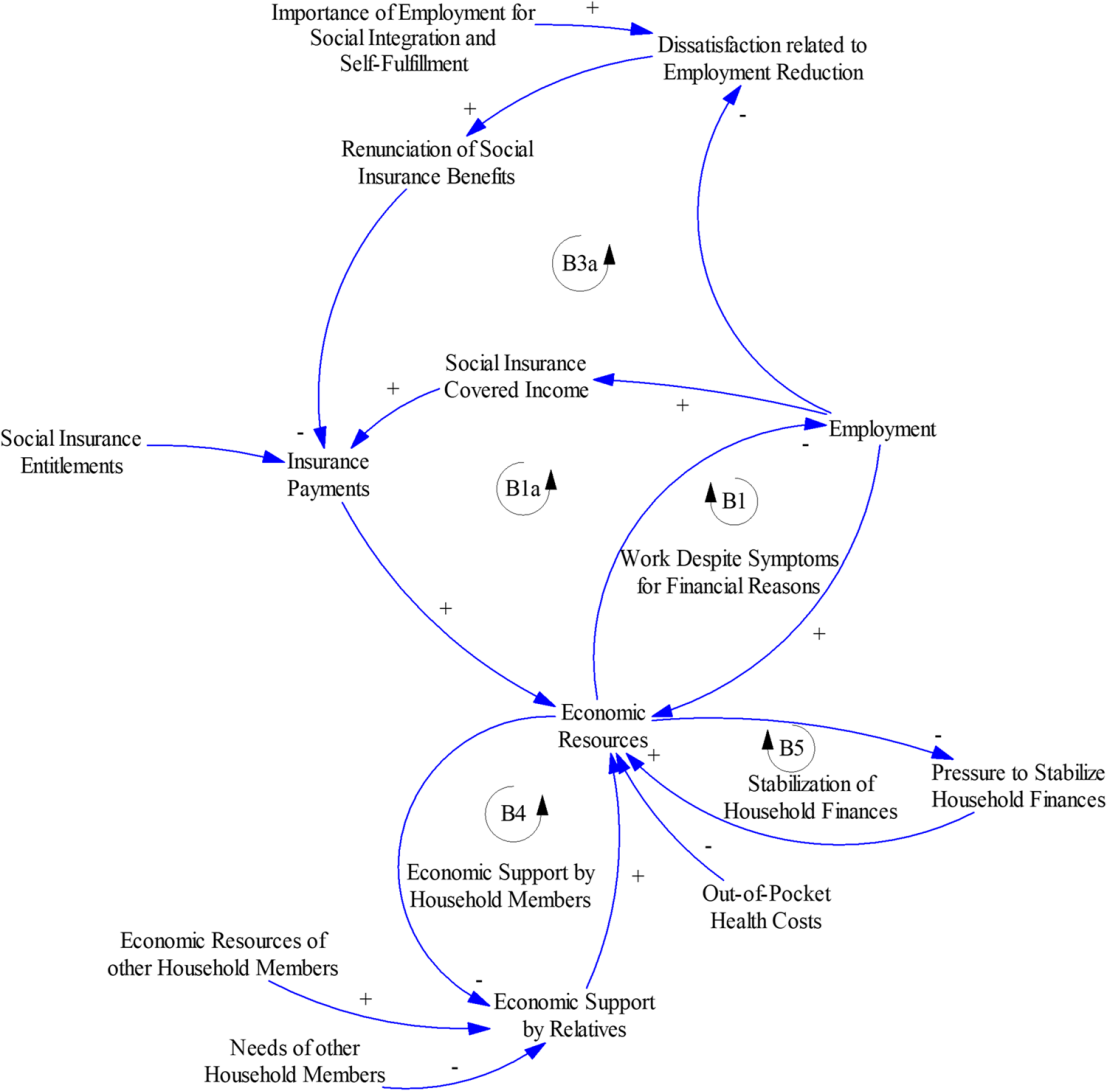
Coping with limited economic resources

This sector of the model is depicted in Fig. 3 and focuses on various strategies used by cancer survivors and their relatives to stabilize their economic resources. The variable *Economic Resources* refers to the household level, at which household members offer mutual economic support.

*Economic Resources* can be diminished directly by *Out-of-Pocket Health Costs* related to a cancer diagnosis: “Often you have to check; if there is no more money, there is no more money. But when one bill arrives or 4 bills of 2,000 francs each, then you have to pay 8,000 francs at the end of the month” (ID01, male, 40 years).

As described by the model, several other mechanisms such as employment reduction contribute to a decrease in *Economic Resources*. The coping strategies used by households to stabilize *Economic Resources* include the balancing mechanisms *B1—Work Despite Symptoms for Financial Reasons* (discussed above), *B4 – Economic Support by Household Members*, and *B5 – Consolidation of Household Finances* (Table 2).

**Table 2:** Narratives for coping with limited economic resources

Model relationships (Fig. 3)	Relevant feedback loop	Participant narrative
*Economic Resources *→* Economic Support by Relatives *→* Economic Resources*	*B4 – Economic Support by Household Members*	Several participants noted that they relied heavily on financial support by their spouses, as in the following example: “If I had lived alone, without a partner, I don't even know how you can manage that. That's a huge question mark for me” (ID03, female, 53 years). Contrary to these examples, we also spoke to people who were single and who lived alone. These people relied on either other family members or friends or donors
*Economic Resources *→* Pressure to Stabilize Household Finances *→* Economic Resources*	*B5 – Stabilization of Household Finances*	A participant with a brain tumor who was no longer capable of working and was therefore unemployed explained that his wife and mother of two toddlers had started working in a new job to sustain the family economically following his diagnosis (ID10, male, 34 years). Another participant avoided going to dentist appointments to reduce expensive out-of-pocket payments (ID06, male, 55 years)
*Employment *→* Social Insurance Covered Income *→* Insurance Payments *→* Economic Resources*	*B1a—Work Despite Symptoms for Financial Reasons*	For several participants, reductions in *Employment* during or after cancer therapy as well as prior to cancer diagnosis contributed to a limitation of *Income Covered by Social Insurance* during the further course of financial toxicity dynamics. These limitations can have long-term impacts on *Insurance Payments*; in one case, which was particularly highlighted by an expert, these losses had effects on financial toxicity that lasted for decades [15]
*Importance of Employment for Social Integration and Self-Fulfilment *→* Dissatisfaction Related to Employment Reduction *→* Renunciation of Social Insurance Benefits*	*B3a – Work Despite Symptoms for Identity and Social Integration*	With respect to *Insurance Payments*, one participant viewed the disability insurance with a critical eye. He perceived it as an underpayment compared to his salary and the effort he invested into self-employment. He was not sure whether he was ready to perceive himself as the sort of person who claims this type of insurance, as he associated it with old age and disability. Receiving this insurance money would therefore not have reduced his psychological burden (ID01, male, 40 years)

Based on these observations, we modeled lower *Economic Resources* as a cause of increases in *Economic Support by Relatives* ( −), which in turn contributes to the stabilization of *Economic Resources* ( +). *Economic Support by Relatives* depends on *Economic Resources of Other Household Members* ( +) and is limited by the economic *Needs of Other Household Members* ( −).

With respect to *B5 – Stabilization of Household Finances*, lower *Economic Resources* increase *Pressure to Stabilize Household Finances* ( −). This variable indicates perceived economic pressures that cause households to either cut spending or increase earnings (based on the provision of financial support by other household members).

The balancing feedback loops B1, B4, and B5 all contribute to the stabilization of *Economic Resources*, at least in the short term. However, these loops can contribute to triggering reinforcing downward spirals. The links between (excessively high) employment burden and reinforcing loops *R1–R3* were discussed above. The links between coping with limited *Economic Resources* and *Care Resources* and further downward spirals are discussed below. Choosing wisely among the various options for coping to avoid potential downward spirals is—according to our model interpretation—key to allow cancer survivors to stabilize their economic resources. However, the available options are limited and depend critically on preexisting economic resources and on, among other factors, *Social Insurance Entitlements*.

*Income Covered by Social Insurance* depends on employment at the time of sick leave or at the time when the disability is diagnosed. *B1a* is a feedback loop that contributes to *B1—Work Despite Symptoms for Financial Reasons*: reduced *Employment* during certain periods that are critical for insurance coverage bring about limited *Insurance Payment* and thus limited *Economic Resources*. Conversely, high insurance coverage critically protects from more restrictive coping options due to limited *Economic Resources* (Table 2, loop B1a).

*B3a* as a variant of *B3 – Work Despite Symptoms for Identity and Social Integration* is modeled as a balancing feedback loop according to which *Dissatisfaction Related to Employment Reduction* and *Renunciation of Social Insurance Benefits* contribute to lower *Insurance Payments*, thus limiting *Economic Resources* and contributing to the maintenance of a higher level of *Employment* (Table 2, loop B3a).

#### Maintaining care resources under economic pressure and burdens

**Fig. 4:**
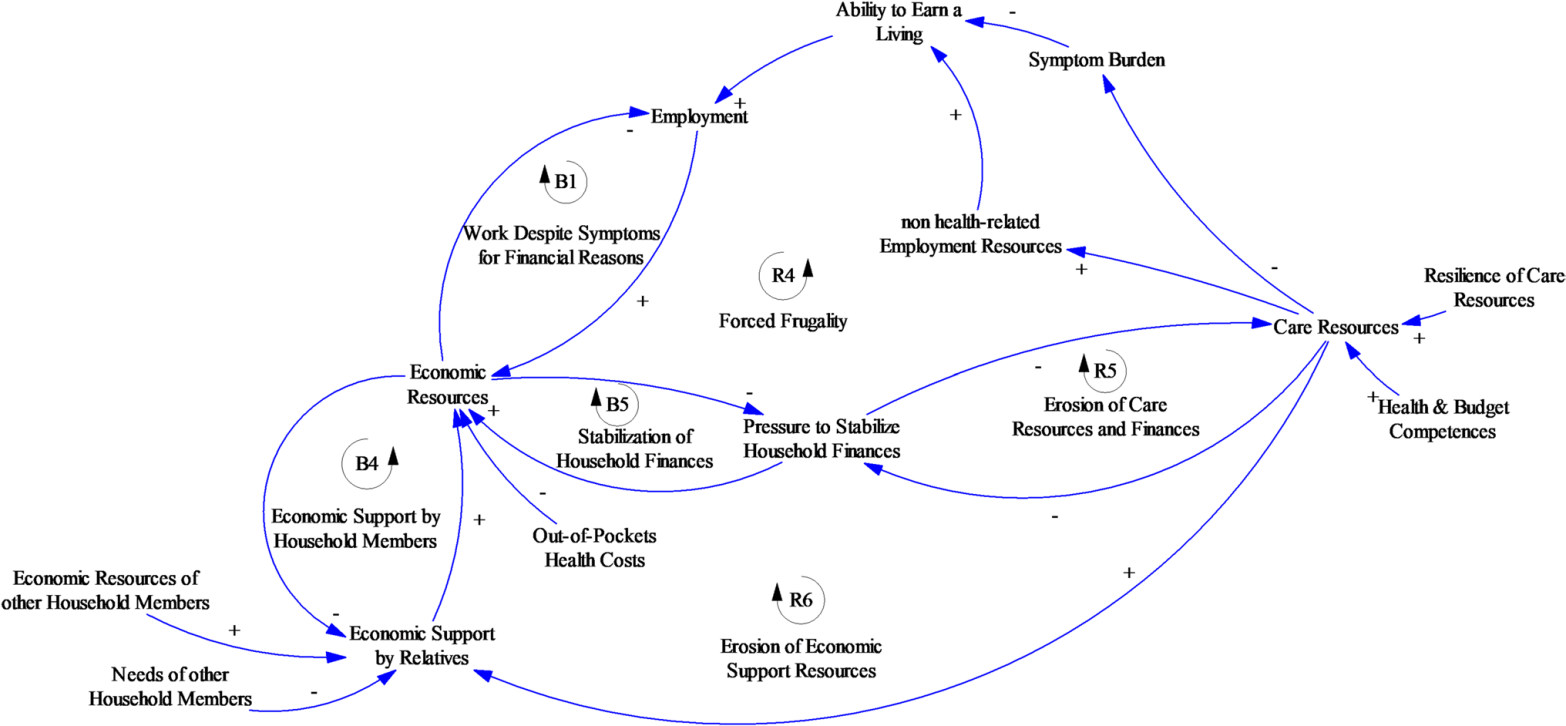
Maintaining care resources under economic pressure

The third model section presented in Fig. 4 focuses on the dynamics associated with maintaining—or eroding—care resources as household finances become strained. We identified the notion of *Care Resources* as a key variable to model the interaction patterns between *Economic Resources*, *Level of Employment*, and *Burdens*, on the one hand, and the resources of the participant’s self, his or her social relationships, and his or her environment on the other. The reinforcing feedback loops *R4—Forced Frugality*, *R5—Erosion of Economic Support Resources*, and *R6—Erosion of Care Resources and Finances* all describe the reinforcing feedback loops that result from this analysis. Disturbances related to the variables involved in this loop tend to be self-reinforcing in the long term unless the critical resources involved in these loops can be supported, maintained, or buffered by the participant with the support of his or her social network.

*R4 – Forced Frugality* can be narrated by using the pressure to consolidate household finances as a starting point. The disturbances introduced in the preceding sections can lead to situations in which pressures to reduce household spending or to increase household earnings continue to increase. These situations require the cancer survivor to weigh the available options carefully to balance his or her financial situation (Table 3, loop R4). The imminent dangers associated with these coping strategies can—on the most abstract level—be described as the risk of the cancer survivor and his or her relatives draining their *Care Resources* by prioritizing their efforts to consolidate household finances.

**Table 3:** Narratives for maintaining care resources under economic pressure

Model relationships (Fig. 4)	Relevant feedback loop	Participant narrative
*Economic Resources *→* Pressure to Stabilize Household Finances *→* Care Resources*	*R4 – Forced Frugality*	One participant decided to leave Switzerland and move to a different country to balance her finances since she perceived the cost of living to be cheaper elsewhere: “Finances are certainly one of the reasons why I'm going back to Germany, quite honestly.” Simultaneously, however, she was willing to leave the country in which she had spent a significant part of her life, thereby leaving behind the social network that had greatly supported her thus far and possibly eroding her *Care Resources* (ID02, female, 52 years)
*Pressure to Stabilize Household Finances *→* Care Resources *→* non health-related Employment Resources*	*R4 – Forced Frugality*	Another example of the possibility of draining one’s *Care Resources* was a situation in which a participant decided to not take her driver’s test to cut costs. The lack of a driver’s license, however, diminished her chances of finding and being able to accept certain jobs (ID04, female, 41 years)
*Pressure to Stabilize Household Finances *→* Care Resources *→* Pressure to Stabilize Household Finances*	*R5 – Erosion of Care Resources*	One participant, a single parent of two small children, decided to employ a daycare service every time he stayed in the hospital to receive cancer treatment (ID11, male, 46 years). However, this service was very expensive, thereby increasing *Pressures to Stabilize Household Finances* and thus, as discussed above, potentially limiting *Care Resources* in other contexts
*Resilience of Care Resources *→* Care Resources *→* Economic Support by Relatives*	*R6 – Erosion of Economic Support Resources*	One partner of a participant was heavily involved in the tasks of organizing his partner’s finances and providing daily support, thereby depleting his own energy. He considered what might happen if he himself were to become ill: “But if she separates from me now or I'm no longer well, then she has a problem” (relative of ID04, female, 41 years)

Limited *Care Resources* potentially contribute, as discussed above, to reinforce or prolong *Symptom Burden*, further limiting cancer survivors’ *Ability to Earn a Living* and thus further draining their *Economic Resources*. Additionally, according to our broad understanding, draining or limiting *Care Resources* can cause *non-health-related employment resources* to be drained or limited (Table 3, R4). Further decreases in *Economic Resources* tend to increase *Pressure to Stabilize Household Finances*—a link that completes the downward spiral.

In addition to R4, *R5 – Erosion of Care Resources* indicates that limited *Care Resources* and *Pressures to Stabilize Household* finances can mutually reinforce each other (Table 3, R5).

*R6 – Erosion of Economic Support Resources* additionally indicates that a loss of *Care Resources* and a loss of *Financial Support by Other Household Members* can reinforce each other. Experts highlighted cases in their practice in which a limitation of resources for self-care, parental care, or mutual care of household members in cases of cancer strained social relationships ultimately led to reduce mutual financial support—for example, due to a divorce following a cancer diagnosis. In our participants’ narratives, we did not find direct evidence of this causal relationship. However, the increased dependence of the partner affected by cancer and the associated strained social relationship were expressed by the relative of one participant (Table 3, loop R6). Based on this evidence, we modeled strained *Care Resources* as limiting *Financial Support by Other Household Members*. As we discussed above, limited *Financial Support by Relatives* leads to diminishing *Economic Resources*, higher *Pressure to Stabilize Household Finance*, and a further drain of *Care Resources*—thus completing the reinforcing spiral.

### Model interpretation

Our analysis of the interactions among the multiple feedback loops included in the model resulted in three necessary conditions for cancer survivors to avoid financial toxicity (Table 4). We justify these conditions in the following subsections (“Ability to adjust employment to symptom burden,” “Ability to stabilize financial resources,” and “Ability to stabilize care resources”). Each condition is justified based on one of the sections of the model (“Coping with cancer and employment,” “Coping with limited economic resources,” and “Maintaining care resources under economic pressure and burdens”). The criteria that qualify a condition as necessary are the interactions among the sectors of the model (Fig. 1). However, our model interpretation does not follow a strictly deductive chain of reasoning based on the model structure. The generalized findings presented here were discussed once again and validated in workshops with experts and against the backdrop of previous theoretical findings.

**Table 4:** Three necessary conditions for cancer survivors to avoid reinforcing the dynamics of financial toxicity

1) Ability to adjust employment to symptom burden	With the support of his or her social network, the cancer survivor can adjust his or her level of gainful employment dynamically such that he or she can cope with both symptom burden and job resource loss
2) Ability to stabilize financial resources	With the support of his or her social network, the cancer survivor can stabilize his or her financial resources despite reductions in paid work and additional expenses
3) Ability to stabilize care resources	With the support of his or her social network, the cancer survivor can stabilize his or her care resources despite reductions in financial resources and higher burdens

Although we formulated the three necessary conditions as separate statements, we should not forget the interactions among them. If we explore the causal relationships in the model, we find that if one of these conditions is violated, some of the resources that are used to secure the others may begin to drain. These mutual relationships are the factor that qualifies each individual condition as necessary to avoid financial toxicity.

#### Ability to adjust employment to symptom burden

The conflicts that many cancer survivors face when coping with the process of reducing, interrupting, and re-entering employment can be illustrated by reference to Odysseus’ navigation between the two threats of Scylla and Charybdis in the Greek mythology (cited from [28]). A reduction in employment can relieve the burden directly caused by paid work activities. However, some cancer survivors experience dissatisfaction with such employment reduction. Thus, reducing employment can but does not necessarily contribute to protection against a reinforcing downward spiral in which health and care resources erode. In some instances, the burden associated with employment reduction is perceived as an even stronger downward spiral. Moreover, working fewer hours can potentially erode job resources—another way in which such a spiral can be reinforced—and reduce economic resources. Insufficient insurance coverage may be prohibitive with respect to longer absences from work.

To navigate this dilemma, cancer survivors can use a variety of resources—if those resources are available. Such resources include the physical resources necessary to work despite symptoms; the psychological resources necessary to cope with one’s limited ability to work or with the psychological pressures caused by work despite one’s symptoms (for example, the use of social and legal resources in the workplace to develop arrangements in the workplace that allow one to adapt the situation to his or her health-related needs); the working skills, personal qualifications, and professional networks necessary to maintain and rebuild one’s non-health-related work resources continuously; and the economic support, social insurance, or preexisting financial reserves necessary to cope with reduced income economically. Over a period of years or, in many cases, decades, cancer survivors must develop wise coping strategies that take into account their social environment to allow them to avoid and counteract potentially reinforcing downward spirals in a timely manner. Their chances of success in this endeavor clearly depend on the preexistence of the mentioned resources as well as on cancer survivors’ awareness of both these resources and the multifaceted nature of downward spirals. To summarize this discussion in light of the first section of the model, we formulate a first necessary condition for avoiding financial toxicity: with the support of his or her social network, the cancer survivor is able to adjust his or her level of gainful employment dynamically such that he or she can cope both with symptom burden and job resource loss (Table 4).

#### Ability to stabilize financial resources

Lower income and higher expenses both put pressure on the household budgets of cancer survivors. Critical resources for successfully stabilizing economic resources include (1) the amount of preexisting economic reserves, (2) the amount of insurance coverage, (3) rights and social support in a preexisting labor contract, and (4) the capability to reduce household expenses and to obtain financial support from other household members. All these resources are coupled with the psychosocial resources that enable the cancer survivor to make use of existing opportunities and rights to obtain financial support and with the capability to maintain and renew these opportunities and rights over a period of years and, in some cases, decades. In this respect, we formulate a second necessary condition for avoiding financial toxicity: with the support of his or her social network, the cancer survivor is able to stabilize his or her financial resources despite a reduction in paid work and the occurrence of additional expenses (Table 4).

#### Ability to stabilize care resources

Cancer survivors and their relatives cope with increased care needs, but the care resources to which they have access to are reduced. Care resources are drained both by the various burdens associated with cancer and by pressures on the household budget; both types of drain have the potential of self-reinforcement. To protect themselves from that reinforcing drain, cancer survivors once again use and maintain a variety of resources, with a particular focus on the resilience of care resources. Care resources are resilient if the cancer survivors and their relatives have the capabilities necessary to “continue, maintain, and repair the life-sustaining web, including their bodies, their selves and their environment” when facing increased burden and pressures “such that they can live in it as well as possible” [27]. Highly resilient care resources potentially counteract high burdens and budget pressure over a long period of time, thus protecting cancer survivors from reinforcing dynamics involving care resources. Aspects of resilience that we particularly highlight in the context of financial toxicity are health and budget competences, which enable the cancer survivor and his or her relatives to make frugal decisions to ensure that their health and well-being are affected as little as possible. However, the longer the physical, psychosocial, or budgetary pressure in question lasts, the higher the strain on care resources and the more care resources are threatened to erode, with the potential of triggering a reinforcing downward spiral. Our third necessary condition to avoid financial toxicity is thus as follows: with the support of his or her social network, the cancer survivor is able to stabilize his or her care resources despite a reduction in financial resources and the imposition of higher burdens (Table 4).

## Discussion

In our study, we examined the circular dynamics of cancer-related financial toxicity by developing a causal loop diagram. We were able to elaborate three necessary conditions for avoiding financial toxicity based on the themes: (1) ability to adjust employment to symptom burden, (2) ability to stabilize financial resources, and (3) ability to stabilize care resources. Our findings confirm the results of previous research, as, e.g., the circular processes underlying the interaction between what we describe as care resources and well-being have already been discussed in a recent review of qualitative studies [5]. In the same review, the circular processes underlying the interactions among increased cost, reduced income and well-being are discussed. In that sense, our findings confirm the results of previous research and complement it by reference to empirical data from Switzerland. We represent these findings on cancer-related financial toxicity in the form of a causal loop diagram. In this representation, reinforcing and balancing feedback loops are presented in a more explicit way than in previous analyses. For the model to remain intelligible, the more explicit representation of feedback loops comes at the cost of a higher level of abstraction for some variables, for example, for care resources. The various aspects of this variable were presented in a more in-depth way in previous research [1]. We were required to make this compromise to avoid a situation in which the number of arrows and variables became too large to remain intelligible.

### Practical model applicability

Raising awareness of the phenomenon of cancer-related financial toxicity, strategies to balance financial toxicity, and the ways in which unintended mechanisms can reinforce financial toxicity is key to the prevention and mitigation of financial toxicity. The model presented here can play a role in these educational efforts for all target groups, including professionals, the cancer survivors themselves, and their relatives.

After receiving a cancer diagnosis, the tasks of mastering daily life and coping with related and immediate existential questions are challenging. Although longer-term and indirect consequences and the effects of one’s actions may seem to be less important at this time, cancer survivors, their relatives, and professionals should consider these issues. This problem is not limited to cancer-related financial toxicity but appears in any dynamically complex situation [29]. Circular causal mechanisms in general, such as the reinforcing erosion of economic support resources, can easily remain unanticipated. Once the effects of such mechanisms are noticed, they are hardly reversible. With respect to reinforcing feedback, small causes may, at a later point, have large effects. System dynamics models and causal loop diagrams have the potential to raise awareness of such “hidden feedback” [29].

In the clinical context, there is a need for simple screening instruments for financial toxicity risks. The present model serves as a foundation for the design of a simple questionnaire that, by reference to the three necessary conditions discussed above, contributes to the ability to assess risk factors for financial toxicity while considering cyclical causality and nonlinear effects.

However, such a questionnaire should not be applied blindly but should rather be used or supported by professionals who are aware of the cyclical nature of the problem and the associated balancing and reinforcing mechanisms. We suggest that this model can be used as a basis for training professionals in oncology, oncology nursing, social counseling, and case management. Moreover, we encourage the design of socioeconomic or psychosocial patient education programs based on the model.

### Model validity

During the process of model design, the model was iteratively validated. The validation process was applied to the hierarchical levels of each single variable, each causal link, each feedback loop, the three substructures presented in the results section, and the model boundaries [26]. With respect to causal links, only links that we found empirically to underlie at least one participant’s narrative were included in the model. Simultaneously, for a causal link to be included, necessary conditions were that at least one expert supported its importance with regard to financial toxicity dynamics and that we found theoretical evidence to support its significance. At the level of feedback loops and substructures, necessary conditions included that these factors were appropriate for reconstructing narratives regarding cancer-related financial toxicity dynamics and that this relevance was confirmed by participating experts. Reinforcing and balancing interactions that were found to be of major importance in the narrative of either an expert or a participant and that were not contested by one of the aforementioned exclusion criteria were required to be included in the model. After 11 interviews were conducted, saturation was reached, indicating that interactions of major importance were increasingly subsumed under causal mechanisms that were already included in the model. The criterion for the cessation of validation [26] was that the inclusion of additional evidence did not alter the conclusions drawn from the model, i.e., the three necessary conditions for avoiding financial toxicity.

We can thus conclude that the model is valid in the sense that it allows us to reconstruct the causal structure of narratives pertaining to cancer-related financial toxicity—its balancing and reinforcing mechanisms—for the population covered by the sample selection in a manner that is consistent with the perspective of experts and the current state of research from various disciplinary perspectives.

### Limitations

The model describes the feedback loops that potentially balance or reinforce cancer-related financial toxicity. This description is purely qualitative and based on narratives. Depending on the specifics of individual cases and states in the trajectory of financial toxicity, single cause-effect relationships may be invalid or irrelevant. Each causal relationship can have varying temporal characteristics depending on individual cases. It should be noted that causal relationships are generally nonlinear. While we investigated whether a causal link has positive or negative polarity, a priori, the functional dependence between two variables is unknown. Accordingly, the present model is not suitable to make predictions in individual cases. Conversely, it can be used to identify potentially risky scenarios with respect to financial toxicity in a narrative manner and as a basis for the discursive and formative investigation of individual cases. In this sense, the model does not replace the understanding of cases by oncologists, oncology nurses, or social workers but rather supports and enhances that understanding. Similarly, the model can complement the personal experiences and knowledge of cancer survivors and their relatives.

#### Restrictions related to sample selection

The sample was recruited from oncology departments and social counseling services specializing in cancer-related financial toxicity in the eastern part of Switzerland. All participants thus had some level of support from professional services that were aware of their financial toxicity issues. The most serious limitation resulting from sample selection is the dependence of financial toxicity on the financial mechanisms associated with health provision and social security in Switzerland and on legislation pertaining to the protection of employment contracts, which varies internationally. Nevertheless, we hypothesize that while operational details vary across countries, a great deal of the interplay between balancing and reinforcing mechanisms remains valid independent of the specific country in question. This hypothesis is supported by existing international research and review articles as discussed above, which addresses all the topics and many of the interactions described here. Even if this previous research is not presented in the specific form of a causal loop diagram and does not highlight balancing and reinforcing mechanisms, it nevertheless discusses similar concepts and interactions in different national contexts. However, the transferability of these research results to other country-specific contexts remains a hypothesis that awaits empirical confirmation.

#### Restrictions with respect to the method

As many variables interact in cyclical, nonlinear, and delayed relationships, each case’s development over time as observed empirically can be reconstructed in more than one single way. The manner in which these feedback loops were reconstructed in the model was subjective and based on the perspectives of the participants, the experts involved in the study, and the researchers. While the methodological study design allowed us to triangulate these perspectives, other, more convincing ways of reconstructing cancer-related financial toxicity dynamics may exist.

## Conclusions

In this article, we present a causal loop diagram that describes the interplay between the feedback mechanisms that either balance or reinforce cancer-related financial toxicity (Figs. 1, 2, 3 and 4). This causal loop diagram is a conceptual model that explicates this interplay qualitatively. Based on the model, we developed three necessary conditions for cancer survivors to avoid financial toxicity (Table 4).

Three model sections, all of which are mutually interlinked, focus on the tasks of coping with cancer and employment, coping with limited economic resources, and maintaining care resources while facing economic pressure. Coping with limited economic resources puts stress on personal burden due to the need to maintain employment at a high level as well as on social resources due to the need to support the cancer survivor economically and stabilize household finances. The model describes the ways in which these coping strategies can be undermined by unintended feedback loops that can reinforce the problem of financial toxicity. These strategies involve psychological and physical burdens that erode both economic resources as well as social and instrumental resources. These resources are lacking due to financial coping or cancer burden. This lack of economic resources leads to a downward spiral.

The model is based on the triangulation of cancer survivors’ narratives with the perspectives of experienced professionals and perspectives drawn from interdisciplinary research. The model has potential as a useful tool for raising awareness of hidden and “invisible” feedback for the purposes of both professional training and patient education. It can be used to identify potentially risky scenarios with respect to financial toxicity in a narrative way and can serve as a basis for a discursive and formative investigation of individual cases. In this sense, the model does not replace the professional experience of oncologists, oncology nurses, or social workers as well as the personal experience and knowledge of cancer survivors and their relatives but rather supports and informs that experience and knowledge. Moreover, an important limitation of the model is its inappropriateness with respect to predicting the outcomes of individual cases. While we hypothesize that the model structure is valid beyond the study context of Switzerland, empirical verification of this hypothesis is yet to be conducted. With respect to its application, implementation research on the ways in which the model can be used in clinical and social counseling contexts represent avenues for future research.

### Supplementary Information

Below is the link to the electronic supplementary material.

Supplementary file1 (TIF 782 KB)

## Data Availability

Data files are available from the corresponding author upon reasonable request.

## References

[1] Fitch MI (2008). Supportive care framework. Can Oncol Nurs J.

[2] Altice CK, Banegas MP, Tucker-Seeley RD, Yabroff KR (2017) Financial hardships experienced by cancer survivors: a systematic review. J Natl Cancer Inst 109. 10.1093/jnci/djw205 10.1093/jnci/djw205 PMC6075571 27754926

[3] Zafar SY (2016) Financial toxicity of cancer care: it’s time to intervene. J Natl Cancer Inst 108. 10.1093/jnci/djv370 10.1093/jnci/djv370 26657334

[4] Lueckmann SL, Schumann N, Hoffmann L (2020). ‘It was a big monetary cut’ - a qualitative study on financial toxicity analysing patients’ experiences with cancer costs in Germany. Health Soc Care Commun.

[5] Fitch MI, Sharp L, Hanly P, Longo CJ (2021). Experiencing financial toxicity associated with cancer in publicly funded healthcare systems: a systematic review of qualitative studies. J Cancer Surviv.

[6] Witte J, Mehlis K, Surmann B (2019). Methods for measuring financial toxicity after cancer diagnosis and treatment: a systematic review and its implications. Ann Oncol: Off J Eur Soc Med Oncol.

[7] Pearce A, Tomalin B, Kaambwa B (2019). Financial toxicity is more than costs of care: the relationship between employment and financial toxicity in long-term cancer survivors. J Cancer Surviv.

[8] Yeager KA, Zahnd WE, Eberth JM (2022). Financial navigation: staff perspectives on patients’ financial burden of cancer care. J Cancer Surviv.

[9] Gordon LG, Merollini KMD, Lowe A, Chan RJ (2017). A systematic review of financial toxicity among cancer survivors: we can’t pay the co-pay. Patient.

[10] Smith GL, Lopez-Olivo MA, Advani PG (2019). Financial burdens of cancer treatment: a systematic review of risk factors and outcomes. J Natl Compr Cancer Netw:JNCCN.

[11] Lu L, Gavin A, Drummond FJ, Sharp L (2021). Cumulative financial stress as a potential risk factor for cancer-related fatigue among prostate cancer survivors. J Cancer Surviv.

[12] Hastert T, Banegas MP, Hamel LM (2019). Race, financial hardship, and limiting care due to cost in a diverse cohort of cancer survivors. J Cancer Survivorship: Res Pract.

[13] Davis ME, Fugett S (2018). Financial toxicity: limitations and challenges when caring for older adult patients with cancer. Clin J Oncol Nurs.

[14] Journal of Cancer Survivorship. Aims and Scope. https://www.springer.com/journal/11764/aims-and-scope?gclid=CjwKCAiAjs2bBhACEiwALTBWZaMjaS_cosPnHaTTW6lJWmYrWe-wnf2H4iTOU2jQJ9S14brrdpHNshoCcdoQAvD_BwE. Accessed 17 Nov 2022.

[15] Kobleder A, Richle E, Müller M (2020). Gesundheitsrisiko Geld – Sozioökonomische Auswirkungen einer Krebserkrankung.

[16] Head B, Harris L, Kayser K, Martin A, Smith L (2018). As if the disease was not enough: coping with the financial consequences of cancer. Support Care Cancer : Off J Multinatl Assoc Support Care Cancer.

[17] Banegas MP, Schneider JL, Firemark AJ (2019). The social and economic toll of cancer survivorship: a complex web of financial sacrifice. J Cancer Surviv.

[18] Céilleachair AÓ, Costello L, Finn C (2012). Inter-relationships between the economic and emotional consequences of colorectal cancer for patients and their families: a qualitative study. BMC Gastroenterol.

[19] Richardson GP (1991) Feedback thought in social science and systems theory. University of Pennsylvania Press. https://books.google.ch/books?id=MOQCzgEACAAJ

[20] Sterman J (2000) Business Dynamics: Systems Thinking and Modeling for a Complex World. McGraw-Hill Higher Education. Irwin/McGraw-Hill

[21] Darabi N, Hosseinichimeh N (2020). System dynamics modeling in health and medicine: a systematic literature review. Syst Dyn Rev.

[22] Lane DC (1999). Social theory and system dynamics practice. Eur J Oper Res.

[23] Stave K (2010). Participatory system dynamics modeling for sustainable environmental management: observations from four cases. Sustainability.

[24] Andersen DF, Richardson GP (1997). Scripts for group model building. Syst Dyn Rev.

[25] Flick U (2018). An introduction to qualitative research.

[26] Groesser SN, Schwaninger M (2012) Contributions to model validation: hierarchy, process, and cessation. Syst Dyn Rev 157–81. 10.1002/sdr.1466

[27] Tronto JC (1990) Towards a feminist theory of caring. In: Abel EK, editor. Circles of care. Work and identity in women’s lives. Albany, N.Y.: State University of New York Press pp. 36–54.

[28] Lane DC, Husemann E (2008). Steering without Circe: attending to reinforcing loops in social systems. Syst Dyn Rev.

[29] Scheidegger A, Müller M. Arrer E. Fringer A (2020) Das dynamische Modell der Angehörigenpflege und -betreuung. Zeitschrift für Gerontologie und Geriatrie 318–26. 10.1007/s00391-019-01574-8 10.1007/s00391-019-01574-8 31278488

