# Supplementary figures and images for: Financial toxicity among cancer survivors: a conceptual model based on a feedback perspective
Source: Support Care Cancer. 2023 Oct 7;31(10):618. doi: 10.1007/s00520-023-08066-x (PMC10560155; doi:10.1007/s00520-023-08066-x)

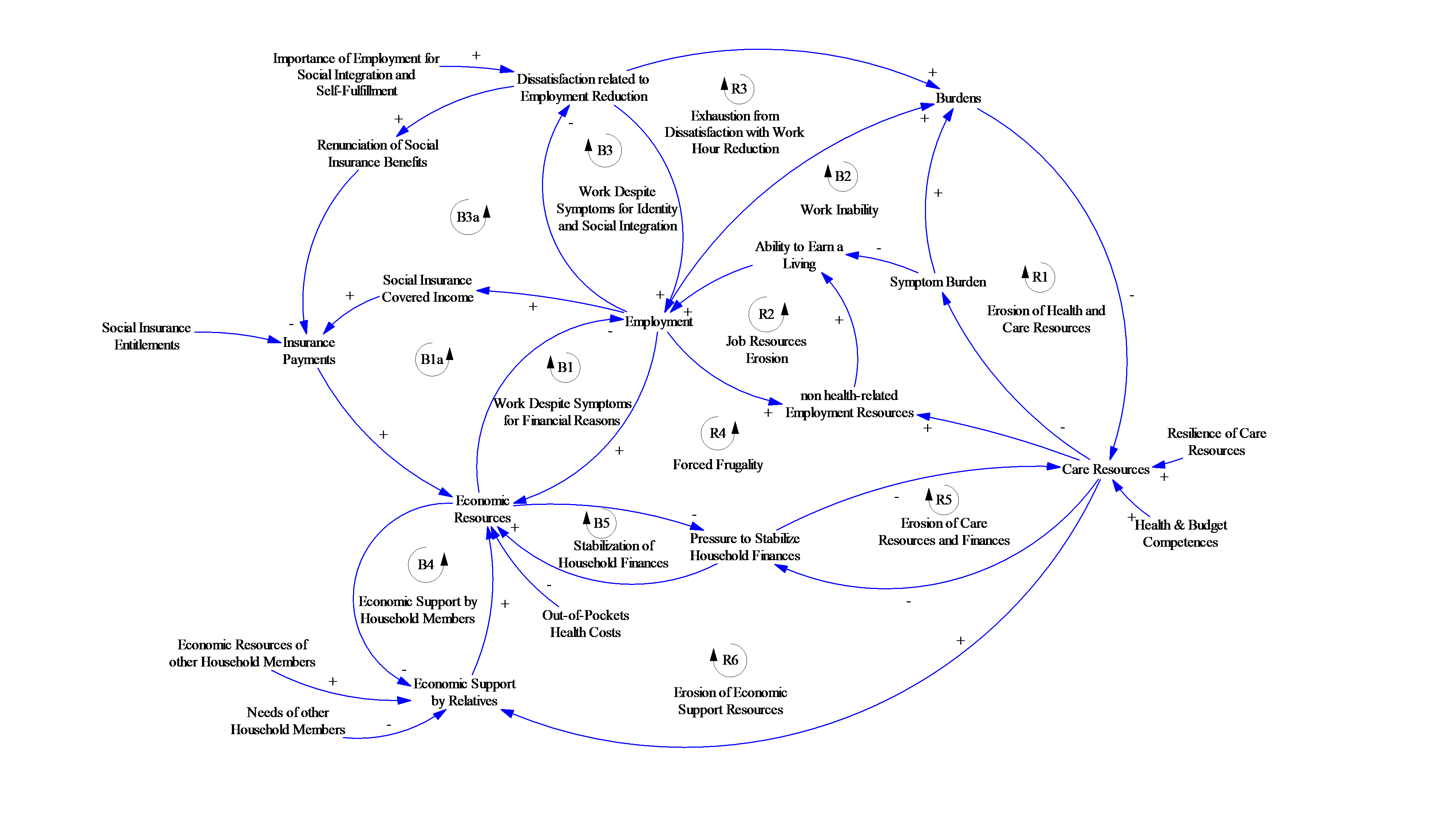

Supplement: Supplementary file 1 — Supplementary file1 (TIF 782 KB) [file 520_2023_8066_MOESM1_ESM.tif]
